# Biological Role and Clinical Implications of microRNAs in BRCA Mutation Carriers

**DOI:** 10.3389/fonc.2021.700853

**Published:** 2021-09-06

**Authors:** Chiara Tommasi, Benedetta Pellegrino, Daniela Boggiani, Angelica Sikokis, Maria Michiara, Vera Uliana, Beatrice Bortesi, Francesco Bonatti, Paola Mozzoni, Silvana Pinelli, Anna Squadrilli, Maria Vittoria Viani, Diana Cassi, Giuseppe Maglietta, Marco Meleti, Antonino Musolino

**Affiliations:** ^1^Medical Oncology and Breast Unit, University Hospital of Parma, Parma, Italy; ^2^Department of Medicine and Surgery, University of Parma, Parma, Italy; ^3^GOIRC (Gruppo Oncologico Italiano di Ricerca Clinica), Parma, Italy; ^4^Medical Genetics Unit, University Hospital of Parma, Parma, Italy; ^5^Dental School, Department of Medicine and Surgery, University of Parma, Parma, Italy; ^6^Unit of Dentistry and Oral-Maxillo-Facial Surgery, Surgical, Medical and Dental Department of Morphological Sciences related to Transplant, Oncology and Regenerative Medicine, University of Modena and Reggio Emilia, Modena, Italy; ^7^Research and Innovation Unit, University Hospital of Parma, Parma, Italy

**Keywords:** miRNAs, breast cancer, nutriepigenomics, BRCA1/2 mutations, breast cancer risk

## Abstract

Women with pathogenic germline mutations in *BRCA1* and *BRCA2* genes have an increased risk to develop breast and ovarian cancer. There is, however, a high interpersonal variability in the modality and timing of tumor onset in those subjects, thus suggesting a potential role of other individual’s genetic, epigenetic, and environmental risk factors in modulating the penetrance of BRCA mutations. MicroRNAs (miRNAs) are small noncoding RNAs that can modulate the expression of several genes involved in cancer initiation and progression. MiRNAs are dysregulated at all stages of breast cancer and although they are accessible and evaluable, a standardized method for miRNA assessment is needed to ensure comparable data analysis and accuracy of results. The aim of this review was to highlight the role of miRNAs as potential biological markers for BRCA mutation carriers. In particular, biological and clinical implications of a link between lifestyle and nutritional modifiable factors, miRNA expression and germline *BRCA1* and *BRCA2* mutations are discussed with the knowledge of the best available scientific evidence.

## Introduction

Breast cancer (BC) is the most common cancer in women, as the cumulative risk of developing a BC during all life is calculated to be about 1 case every 8 women worldwide (1) (2). BC is also the principal cause of cancer death among women worldwide accounting for 25% of cancer cases and 15% of cancer-related deaths (3).

Hereditary breast cancer accounts for about 5-10% of all breast cancers (BCs) and is associated with an increased risk of ovarian cancer (4, 5).

Hereditary Breast and Ovarian Cancer syndrome (HBOC) is related, in about 50% of cases, to pathogenic germline mutations of *BRCA1* and *BRCA2* genes (6, 7). *BRCA1/2* genes are onco-suppressors involved in homologous recombination repair (HRR) of DNA double-strand breaks (DSBs) and maintenance of genome stability (8). Women who inherit *BRCA1/2* mutations have a lifetime risk to develop breast and ovarian cancer of 45-60% and 10-59%, respectively (9) (10). In these cases, viable prevention strategies include intensive radiologic surveillance, chemoprevention, and prophylactic surgery of breasts and ovaries (11).

Although breast and ovarian cancer risk increases considerably, not all women with *BRCA1/2* mutations develop a neoplasm. There is a high interpersonal variability in the modality and timing of tumor onset in BRCA-mutated subjects, thus suggesting a potential role of other genetics, epigenetics, or environmental individual risk factors in modulating the penetrance of *BRCA1/2* germline mutations (12).

Transcribed, non-coding RNAs (ncRNAs) do not encode for proteins and have a specific biological function (13). NcRNAs have various transcripts’ lengths: short ncRNAs are <50 nucleotides (nt), as well as microRNAs (miRNAs); midsize ncRNAs include the ncRNAs between 50 nt and 200 nt. Finally, long ncRNAs (lncRNAs) have a length over 200 nt (13–15).

MiRNAs are involved in post-transcriptional, epigenetic modification of DNA expression (16). They are readily detectable in tissue and blood samples (17), saliva (18) or urine (19). While it is difficult to establish cause-and-effect relationships, several studies indicate that some miRNA expression patterns may be associated with: i) increased breast/ovarian cancer risk; ii) some modifiable nutrition/lifestyle risk factors; iii) *BRCA1/2* mutations (12, 17). Gene panels, which simultaneously evaluate whole miRNAs, are able to identify different miRNA expression profiles between healthy women, women with sporadic BC and women with BRCA-mutated BC (19, 20).

Based on these considerations, the aim of this review is to highlight the role of miRNAs as potential biomarkers for BRCA mutation carriers. Biological and clinical implications of a link between lifestyle and nutritional modifiable factors, miRNA expression and germline *BRCA1* and *BRCA2* mutations are here discussed with the knowledge of the best available scientific evidence.

## Mechanistic Insights of the Interaction of miRNAs With BRCA Genes

MiRNAs are critical regulators of the transcriptome over a number of different biological processes and they may behave as onco-suppressors and onco-promoters (21). MiRNAs can post-transcriptionally suppress gene expression by binding to the 3′-untranslated region (UTR) of messenger RNA (mRNA) (22). However, miRNA interactions with other regions, which include the 5′-UTR, coding sequence, and gene promoters, have also been described (22, 23). Moreover, miRNAs have been shown to trigger gene expression under certain condition (21). Recent studies have demonstrated that miRNAs are transferred between various subcellular compartments to regulate both translation and transcription (21) (22).

**Figure 1:**
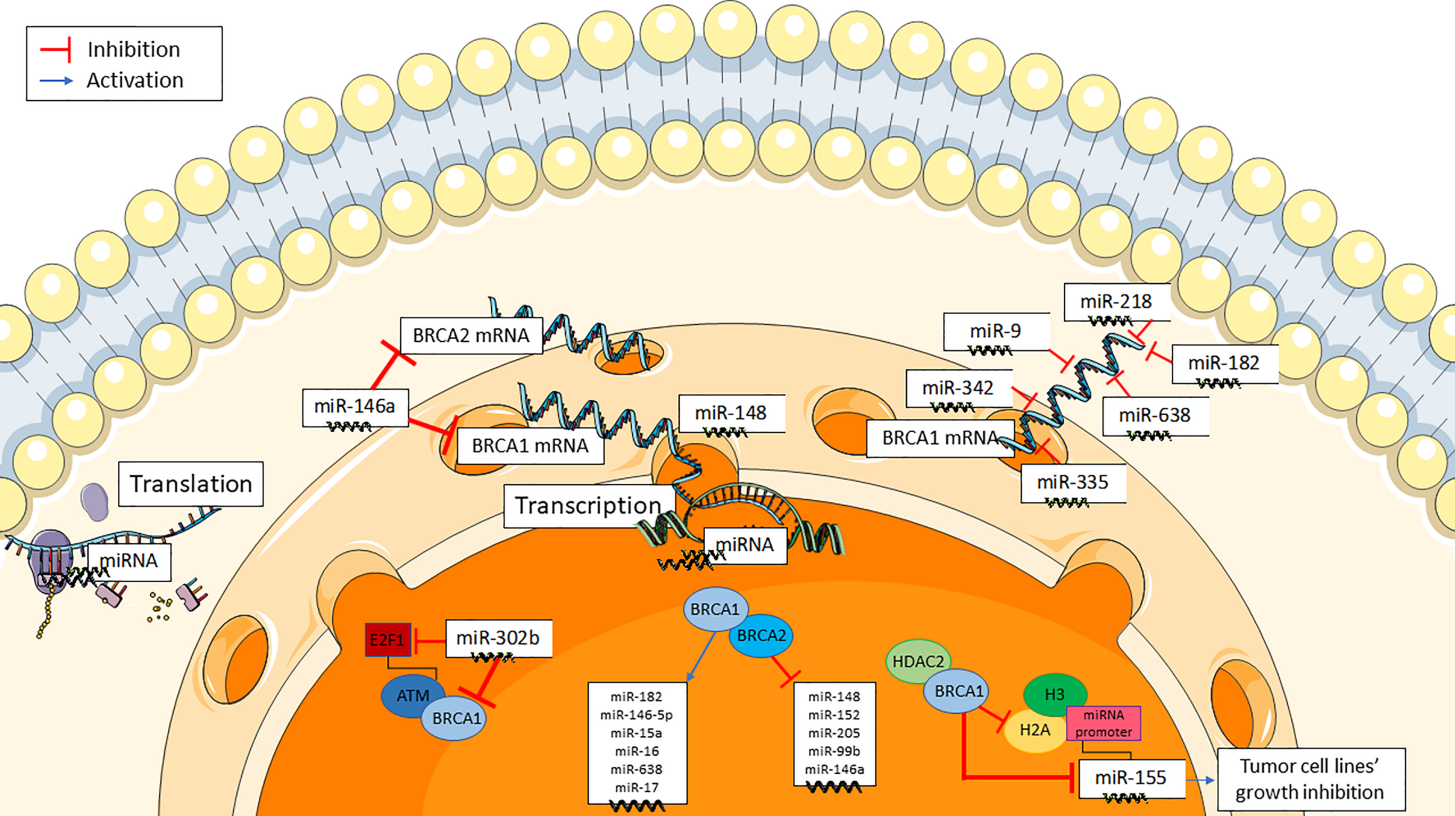
Mechanistic insights of the interaction of miRNAs with BRCA genes.

*BRCA1/2* gene expression can be altered by miRNAs, in addition to deletion or mutation, in a BRCAness-like phenomenon (**Figure 1****)** (21). E2F1, a G1/S transition regulator, is targeted by miR-302b in breast cancer cell lines. MiR-302b, by negatively regulating E2F1, downregulates ATM, the principal cellular sensor of DNA damage, that phosphorylases and actives BRCA1. As a result, miR-302b indirectly impairs BRCA1 function (23). Furthermore, various studies have evaluated some miRNAs targeting BRCA genes in breast cancer. MiR-146a binds to the 3′-UTRs of BRCA1and BRCA2 mRNAs, thus negatively modulating their expression. Interestingly, the binding capacity of miR-146a seems to be dependent from some of its gene polymorphisms (24). In human tumor xenografts, miR-9 has been found to bind to the 3’-UTR of BRCA1 mRNA, downregulate BRCA1 expression, and enhance cancer cell susceptibility to DNA damage (25). Similar findings have been reported for miR-182 (26), miR-155 (27), miR-342 (28), miR-335 (29), miR-218 and miR-638 (23).

*BRCA1* and *BRCA2* genes also regulate several miRNAs either by upregulating some of them (i.e. miR-146a, miR-146-5p, miR-182, miR-15a, miR-16, miR-17, and miR-638), or by downregulating some others (i.e. miR-155, miR-152, miR-148, miR-205, miR-146a, and miR-99b) (**Figure 1**) **(**19). Notably, BRCA1 is involved in the epigenetic control of miR-155, which is a well-known proinflammatory and oncogenic miRNA (27, 30). MiR-155 overexpression stimulates while miR-155 knockdown impairs cancer cell growth. BRCA1 targets the miR-155 promoter, thus suppressing its transcription. More precisely, BRCA1 suppresses miR-155 expression through its association with histone deacetylase 2 (HDAC2), which deacetylates histones H2A and H3 on the miR-155 promoter (27). Some moderate-risk BRCA1 variants (e.g. R1699Q) do not affect DNA repair but abrogate the inhibition of microRNA-155 (27).

## Role of miRNAs in Different Breast Cancer Subtypes

Breast cancers are usually grouped into surrogate intrinsic subtypes, defined by routine histology and immunohistochemistry (IHC): luminal A-like tumors are generally low grade, strongly estrogen receptor (ER)/progesterone receptor (PR)-positive, human epidermal growth factor receptor (HER2)-negative and have low proliferation rate. Luminal B-like tumors are ER-positive with variable degrees of ER/PR expression, are higher grade and have higher proliferation rate. HER2-positive tumors are usually high grade, frequently ER/PR-negative, and have high proliferative rate. Triple-negative breast cancers (TNBCs) are high grade, ER/PR-negative, HER2-negative, and have high proliferation rate (1) (2) (3). The expression of numerous miRNAs correlates with different BC subtypes and different prognosis (**Table 1**).

**Table 1:** miRNAs as diagnostic and prognostic biomarkers in breast cancer.

miRNA role/function	Identified miRNAs	miRNA detection tissue	Reference
Overexpressed in Luminal-like BC	miR-29; miR-181a; miR-652; miR-342	Serum	(31)
	miR-100; miR-155; miR-126; miR10a; let-7c; let-7f; miR-217; miR-218; miR-377; miR-520f-520c; miR-18a	Tumor tissue	(32–34)
Overexpressed in HER2-positive BC	miR-21; miR-376b	Serum	(35, 34)
Overexpressed in TNBC	miR-210; miR-146a; miR-146b-5p; miR-10b; miR-18a; miR-135b; miR-93v; miR-299-3p; miR-190; miR-135b; miR-520g; miR-527-518a	Tumor tissue	(34, 36–38)
Overexpression associated with endocrine sensitivity	miR-27a; miR-100; miR-375; miR-342; miR-221/222; let-7f	Tumor tissue	(32, 34)
Overexpression associated with better survival	miR-497; miR10a; miR-126; Let-7b; miR-147b; miR-6715a; miR-324-5p; miR-711; miR-375	Tumor tissue	(32–34, 39–41)
	miR-1258	Tumor tissue/serum	(42)
	miR-92a	Plasma/serum	(43)
Overexpression associated with worse survival	miR-21	Tumor tissue/serum	(29, 43)
	miR-210; miR-205; miR-374a; miR-10b; miR-549a; miR-4501; miR-7974; miR-4675; miR-9; miR-18b; miR-103; miR-107; miR-652; miR-27b-3p	Tumor tissue	(32, 36, 38) (42–44)
Overexpressed in BC compared to HC	miR-1; miR-133a; miR-133b; miR-92a; miR-21; miR-16; miR-27; miR-150; miR-191; miR-200c; miR-210; miR-451; miR-155; miR-195; Let-7b; miR-106b; miR-145; miR-425-5p; miR-139-5p; miR-130a; miR-34a	Tumor/normal tissue/serum	(41–43, 45–50)
	miR-1246; miR-1307-3p; miR-4634; miR-6861-5p; miR-6875-5p; miR-1246; miR-146a; miR-18a	Plasma/serum	(34, 43, 47, 51)
Underexpressed in BC compared to HC	miR-145	Tumor/normal tissue/serum	(41)
	miR-30a	Plasma	(52)
	miR-140-5p; miR-497; miR-199a; miR-484; miR-202; miR-181a (Nassar)	Tumor/normal tissue	(36, 41, 53)

miRNA, microRNA; BC, breast cancer; TNBC, triple negative breast cancer; pts, patients; HC, healthy controls.

Some highly expressed miRNA clusters have been associated with Luminal A-like and Luminal B-like tumors (31, 32). Interestingly, overexpression of miR-100 in basal-like breast cancer cells leads to stemness loss, expression of luminal markers and sensitivity to endocrine therapy (54). Baseline tumor expression of miR-100 has been associated with response to endocrine treatment in patients with ER-positive/HER2-negative breast cancer. In the METABRIC dataset, high expression of miR-100 was observed in luminal A breast cancers with better overall survival (54).

MiR-21 overexpression has been observed in HER2-positive breast cancers, probably because the corresponding gene is located on chromosome 17 and, thus, co-amplifies with HER2 (35, 55). MiR-21 is also an independent prognostic factor associated with early disease relapse and worse disease-free interval (DFI) (56). Furthermore, low miR-497 expression has been observed to be strictly correlated with HER2-positive status and advanced clinical stage (39). In a retrospective case series of tumor tissues and correspondent normal breast tissues, patients with low miR-497 expression had worse 5-year disease-free survival (DFS) and overall survival (OS) than the ones with high miR-497 (39).

Approximately 10-15% of breast tumors are known to be of the TNBC subtype, which is considered to have an aggressive clinical history and shorter survival (20, 31, 57, 58). Up to 29% of TNBC patients harbors somatic mutations or epigenetic downregulation of *BRCA1* and *BRCA2* genes (59). Differential miRNA expression could help predict prognosis in patients with TNBC (36, 37). Accordingly, miR-210 is often up-regulated in TNBC tissues and correlates with unfavorable prognosis (38). High levels of miR-146a and miR-146b-5p have also been described in triple negative tumor samples (60). MiR-155 is commonly down-regulated in TNBC, indeed its overexpression is related with good prognosis (61). Interestingly, some miRNAs may play different prognostic roles depending by molecular subtypes: miR-27 is associated with better OS in ER-positive BC patients, while its upregulation is detrimental in ER-negative ones (44).

Several other miRNAs have been shown to influence the prognosis of BC patients (**Table 1**). The deregulation of 5 metastasis-related miRNAs (miR-21, miR-205, miR-10b, miR-210, and let-7a) observed in a series of 84 primary breast tumors significantly correlated with clinical outcome (32, 62). In a sample of 81 postmenopausal, ER-positive BC patients, a higher tumor expression of miR-126 and miR-10a was associated with longer relapse-free survival (RFS) (33). The expression of let-7b in tumor tissues of 80 breast cancer patients was inversely associated with lymph node involvement, OS and RFS (40). MiR-374a expression was significantly elevated in the primary tumors of 33 patients with metastatic disease in comparison with that observed in primary tumors of 133 patients with no evidence of distant metastasis (63). Decreased levels of miR-92a and increased tumor levels of miR-21 were associated with higher tumor stage and presence of lymph node metastases (43). According to other reports, the downregulation of miR-1258 was associated with positive lymph node involvement, later clinical stage, and poor prognosis (42).

## Role of MiRNAs in Breast Cancer Risk and Detection

Several studies focused on identifying either individual or groups of miRNAs to be used for prediction of BC risk and for BC detection (**Table 2**). The most studied miRNAs were analyzed in plasma/serum and tumor tissues of BC patients in comparison with normal controls (41). However, those studies were conducted in different ethnic groups and used different experimental methodology (43). Some studies detected miRNAs in breast tissues using distinct platforms (real-time quantitative reverse transcription PCR [qRT-PCR], sequencing, microarray) followed by validation in serum/plasma (41, 34). Others produced array panels on plasma samples followed by verification using qRT-PCR (47) or started with qRT-PCR on tissues with subsequent serum qRT-PCR (41). One of the limitations of the previously mentioned platforms is their restriction to known miRNAs. Next-generation sequencing (NGS) technologies provide novel approaches for identification of new miRNAs and confirmation of known ones (34).

**Table 2:** Diagnostic parameters to evaluate breast cancer diagnostic ability of individual and combined studied miRNAs.

miRNA	Sensitivity	Specificity (%)	PPV (%)	NPV (%)	DA (%)	Reference
miR-21	73	81	76	78	77	(50, 64–67)
miR-155	78	75	78	75	77	(50, 64–67)
miR-23a	78	75	88	44	68	(50, 64–67)
miR-130a	83	78	83	78	81	(50, 64–67)
miR-145	78	91	78	91	83	(50, 64–67)
miR-425-5p	70	100	70	100	81	(50, 64–67)
miR-139-5p	76	96	76	95	83	(50, 64–67)
miR-451	73	72	73	72	73	(50, 64–67)
miR-200a	69	62	NR	NR	70	(50, 64–67)
miR-200c	71	67	NR	NR	74	(50, 64–67)
miR-141	68	70	NR	NR	74	(50, 64–67)
miR-10b	NR	NR	NR	NR	85	(50, 64–67)
miR-181a	NR	NR	NR	NR	82	(50, 64–67)
miR-106b	NR	NR	NR	NR	89	(50, 64–67)
miR-34a	85	70	93	70	81	(50, 64–67)
miR-200b + miR-429	77	63	NR	NR	75	(50, 64–67)
miR-145 + miR-425-5p	78	95	78	95	84	(50, 64–67)
miR-21 + miR-23a	95	66	95	66	82	(50, 64–67)
miR-21 + miR-130a	88	78	88	78	84	(50, 64–67)
miR-21 + miR-23a +miR-130a	93	78	93	78	86	(50, 64–67)
miR-145 + miR-139-5p +miR-130a	95	86	95	86	92	(50, 64–67)
miR-145 +miR-139-5p +miR-130a + miR-425-5p	97	91	97	90	95	(50, 64–67)
miR-1246 + miR-206 + miR-24 + miR-373	98	96	NR	NR	97	(50, 64–67)

PPV, positive predictive value; NPV, negative predictive value; DA, diagnostic accuracy; NR, not reported.

In a recent study, miRNAs from paired breast tumors, normal tissue, and serum samples of 32 patients were profiled; serum samples from healthy individuals (n = 22) were also used as controls. Twenty miRNAs including miR-21, miR-10b, and miR-145 were found to be differentially expressed in breast cancers. Only 7 miRNAs were overexpressed in both serum and tumors, thus indicating that miRNAs may be selectively released into the serum. MiR-92a, miR-1, miR-133a, and miR-133b were identified as the most significant diagnostic serum markers (45). A combination of 5 serum miRNAs (miR-1307-3p, miR-1246, miR-6861-5p, miR-4634, and miR-6875-5p) were also found to detect breast cancer patients among healthy controls (53).

MiR-21, one of the most common miRNAs in human cells, has been investigated in different diseases including cardiovascular diseases as well as cancers (46). Studies have demonstrated that miR-21 plays an oncogene function in breast cancer by targeting tumor suppressor genes which include programmed cell death 4 (PDCD4), tropomyosin 1 (TPM1), and phosphatase and tensin homolog (PTEN) (68). The diagnostic role of high plasma levels of both miR-1246 and miR-21 was demonstrated by analyzing the contents of circulating exosomes, which are secretory microvesicles that selectively enclose miRNAs (69). Exosomes were collected from the conditioned media of human BC cell lines, murine plasma of patient-derived xenograft models (PDX), and human plasma samples. MiR-21 and miR-1246 were selectively enriched in human BC exosomes and significantly increased in the plasma of BC patients (69). Other studies have suggested a key role for miR-21 in discriminating healthy individuals from BC patients. Overexpression of circulating miR-21 and miR-146a were significantly higher in plasma samples of BC patients, when compared to controls (51). Serum levels of miR-16, miR-21, miR-155, and miR-195 were observed to be higher in stage I BC patients in comparison with unaffected women (51).

Nine miRNAs (miR-16, miR-21, miR-27a, miR-150, miR-191, miR-200c, miR-210, miR-451 and miR-145) were observed to be deregulated in both plasma and tumor tissues from BC patients (47). A validation cohort study reported that a combination of miR-145 and miR-451 was the best biomarker in discriminating breast cancer from healthy controls and other tumor types (47).

The expression of 10 miRNAs (measured by qRT-PCR) has been evaluated in 48 tissue and 100 serum samples of patients with primary BC and in 20 control samples of healthy women (43). The level of miR-92a was significantly lower, while miR-21 was higher in tissue and serum samples of BC patients in comparison with controls. The same expression levels correlated with tumor size and lymph node-positive status (43).

MiR-30a has also been studied as diagnostic biomarker of breast cancer. The median plasma levels of miRNA-30a were significantly lower in a sample of 100 patients with preoperative breast cancer than in 64 age-matched and disease-free controls (52). MiR-497 and miR-140-5p have been shown to be down-regulated in breast tumor samples compared to noncancerous breast tissues, while let-7b seems to be upregulated in BC specimens compared to benign breast diseases (38, 46, 68). Validation of these data in serum or plasma samples is missing.

Another interesting approach is to use miRNAs to unveil BC patients with lymph node involvement (overcoming the prognostic role of the axillary dissection) or to identify patients with metastatic disease. Levels of circulating miR-1258 decreased (42), while miR-10b and miR-373 levels increased (70) in a series of BC patients with lymph nodes metastasis in comparison with non-metastatic patients (70). Furthermore, tumor tissue expression of miR-140-5p decreased in the sequence from stage I to III breast cancer, and was lower in breast tumors with lymph node involvement in comparison with ones without metastasis (71).

Receiver operating characteristics (ROC) analyses have been conducted to evaluate breast cancer diagnostic ability of miRNAs (50, 64–67). Specificity, sensitivity, positive predictive value (PPV), negative predictive value (NPV), and diagnostic accuracy for the most studied individual and combined miRNAs are reported in **Table 2**.

## Role of MiRNAs in BRCA Mutation Carriers

Breast cancer susceptibility in *BRCA1/2* mutation carriers may be related to the aberrant expression of certain miRNA clusters (**Table 3**) (78). MiR-3665, miR-3960, miR-4417, miR-4498, and let-7 have been observed to be overexpressed in women with BRCA*-*mutated breast tumors (75). Conversely, the downregulation of miR-200c has been reported in BRCA-mutated, TNBC (74).

**Table 3:** miRNAs and germinal BRCA mutations.

miRNA role/function	Identified miRNAs	miRNA detection tissue	Reference
Upregulated by wild-type BRCA1	miR-182; miR-146-5p; miR-15a; miR-16; miR-638; miR-17	Tumor/normal tissue/serum	(19)
Downregulated by wild-type BRCA1	miR-148; miR-152; miR-205; miR-99; miR-146a	Tumor/normal tissue/serum	(19, 72)
Combined expressions of miRNAs distinguish between BRCA-mutated and BRCA wild-type BC	miR-142-3p; miR-505; miR-1248; miR-181a-2; miR-25; miR-340	Tumor/normal tissue	(72)
	miR-627; miR-99b; miR-539; miR-24; miR-331; miR-663a; miR-362; miR-145	Tumor/normal tissue	(73)
Downregulated in BRCA-mutated BC	miR-155; let-7a; miR-335	Tumor tissue	(27, 29, 61)
	miR-200c	Cell culture	(74)
Overexpressed in BRCA*-*mutated BC	miR-3665; miR-3960; miR-4417; miR-4498; let-7	Tumor tissue	(75)
Regulate BRCA1 expression in BC	miR-342; miR-182; miR-335; miR-146a, miR-146b-5p; miR-182	Tumor tissue	(26, 28, 60, 76)
Restores HRR and genomic stability in *BRCA2*-mutated cancers	miR-493-5p	Tumor tissue	(77)

miRNA, microRNA; BC, breast cancer; HRR, Homologous Recombination Repair.

Some studies have also shown that genetic polymorphisms in the gene codifying for miR-146a were associated with early onset in familial cases of breast and ovarian tumors (24). Different expression patterns of six miRNAs (miR-505*, miR-142-3p, miR-1248, miR-181a-2*, miR-340*, and miR-25*) have been found to distinguish between BRCA-mutated and BRCA wild-type (BRCAwt) breast tumors with an accuracy of 92% (24,72). Similarly, a miRNA expression analysis using NanoString technology was performed on BRCA-mutated and sporadic, BRCAwt breast tumor tissues. Eight miRNAs (miR-539, miR-627, miR-99b, miR-24, miR-663a, miR-331, miR-362, and miR-145) were differentially expressed in hereditary breast cancers (73). Let-7a and miR-335 are tumor suppressor miRNAs that can impair both tumorigenesis and metastasis. Let-7a and miR-335 expression levels were significantly downregulated in cancers of patients with BRCA mutations in comparison with tumors of BRCAwt subjects (29, 76).

BRCA-mutated tumors are *in vitro* and *in vivo* sensitive to DNA-damaging agents (such as platinum salts) and to poly-ADP ribose polymerase (PARP) inhibitors (PARPi) (8, 79). Up to 29% of patients with TNBC harbor somatic mutations or epigenetic downregulation of *BRCA1* and *BRCA2* genes, thus being sensitive to platinum salts and PARPi (79). MiR-146a, miR-146b-5p and miR-182 downregulate BRCA1 protein expression and some reports have shown that different expression of miR-182 in breast cancer cell lines affects their sensitivity to PARPi (26, 60). Furthermore, other studies have reported that the over-expression of miR-493-5p restores genomic stability of *BRCA2*-mutated/depleted leading to acquired resistance to PARPi/platinum salts (77).

## Nutriepigenomics and Breast Cancer Risk

Nutriepigenomics is the study of nutrients and their effects on human health through epigenetic modifications. Numerous studies have suggested that body mass index (BMI), components of food and lifestyle may interfere with miRNA expression, thus affecting tumors’ initiation and progression (72) (29) (**Table 4**).

**Table 4:** miRNAs, body mass index and lifestyle changes.

miRNA role/function	Identified miRNAs	miRNA detection tissue	Reference
Downregulated in BC of obese patients vs. lean subjects	miR-10b	Tumor/normal tissue	(48)
Inversely correlated with BMI in BC survivors	miR-191-5p; miR17- 5p	Serum	(80–82)
Directly correlated with BMI in BC survivors	miR-122-5p		(82)
Overexpressed during diet and exercise intervention in BC survivors	miR-191-5p; miR-122-5p; let-7b-5p; miR-24-3p	Serum	(82)
Underexpressed during diet and exercise intervention in BC survivors	miR-106b; miR-106b_5p; miR-27a-3p; miR-92a-3p	Serum	(49, 81, 82)
Upregulated post lifestyle intervention in responders vs. baseline and vs. nonresponders postintervention.	miR-10a-5p; miR-211-5p; miR-10a-5p	Serum	(81)
Regulate the expression and activity of PPARγ and C/EBP proteins involved in tumor carcinogenesis, adipocyte differentiation and obesity	Let-7b; miR-27; miR-143; miR-31	Tumor/normal tissue	(77, 82–84)

BC, breast cancer; BMI, body mass index; CRP, C-reactive Protein; IL6, interleukin-6; DFS, disease free survival; OS, overall survival; PPARγ, peroxisome proliferator-activated receptor gamma; C/EBP, CCAAT/enhancer-binding family of proteins.

Obesity causes changes in the physiological function of adipose tissue, leading to adipocyte differentiation, insulin resistance, abnormal secretion of adipokines, and altered expression of hormones, growth factors, and inflammatory cytokines. All these factors are involved in the occurrence of several diseases, such as type 2 diabetes mellitus, cardiovascular disease, and various types of cancers (83). Obesity is one of the major risk factors for breast cancer, especially in post-menopausal women. Recent studies have proposed the association between obesity, cancer and up- or down-regulation of some miRNAs (85).

Obesity has been found to reduce expression of miR-10b in primary tumors compared to normal tissue, thus suggesting that the metabolic state of the organism can alter the molecular composition of a tumor (48, 80, 81, 84). Expression levels of miR-191_5p, miR-122-5p and miR-17_5p, which are involved in tumorigenic processes, have been inversely associated with BMI (82). Levels of miR-191-5p significantly increased during a six-month weight-loss intervention (Lifestyle, Exercise, and Nutrition; LEAN trial) in 100 BC survivors (82). Furthermore, the related family member miR-106b_5p, which is up-regulated in breast cancer patients (49, 81), has been found to significantly decrease in response to exercise intervention and diet (82, 85). The nuclear peroxisome proliferator-activated receptor gamma (PPARγ) plays a critical role in the modulation of cellular differentiation, glucose and lipid homeostasis (84). It has been associated with anti-inflammatory activities, and differentiation of preadipocytes into mature adipocytes together with members of the CCAAT/enhancer-binding family protein (C/EBP) family (82). PPARγ is implicated in the pathology of numerous diseases involving cancer and obesity, and altered expressions of miRNAs, such as let-7, miR-27, and miR-143 have been found to regulate the expression and activity of *PPARγ* (81). Interestingly, miR-31 has been found to impair both BC cell proliferation and adipogenesis by directly targeting C/EBP proteins (81).

Dietary elements, seem to have a key role in regulating miRNAs (84, 86, 87) (**Table 5**).

**Table 5:** Dietary elements and expression/regulation of miRNAs.

Elements	On-target effect	Effect on miRNAs	Outcome	Reference
Curcumin (dyferuloylmethane)	Inhibition of Bcl2 protein	Overexpression of miR-15a and miR-16	Inhibition of anti-apoptotic activity in MCF-7 BC cells	(88)
Curcumin (dyferuloylmethane)	Inhibition of MMPs. Reduction of CXCL1/2 protein levels	Overexpression of miR-181b	Reduction of cancer cells invasivity	(89)
Curcumin (dyferuloylmethane)	Reduction of Axl, Slug, CD24 and Rho-A protein levels	Overexpression of miR-34a	Inhibition of EMT in MCF-10F and MDA-MB-231 BC cell lines	(90)
Genistein	Upregulation of PTEN/FOXO3/AKT axis	Underexpression of miR-155; overexpression of miR-23b	Anti-proliferative and pro-apoptotic effects in Hs578t and MDA_MB-435 BC lines	(91)
Rasveratrol	Downregulation of EF1A2 gene expression	Overexpression of miR-663, miR-141, miR-774 and miR-200c	Antiproliferative effect in MCF-7 BC cells; inhibition of CSC phenotype transition	(87, 92)
1,25-D	p53-mediated regulation of PCNA	Overexpression of miR-182	Reduction of cellular stress	(81)
1,25-D	Erα upregulation	Underexpression of miR-489	Antiproliferative effect in ER-positive BC cell lines	(93)
SCFAs acetate (butyrate, propionate)	Activation of FFARs	Overexpression of miR-31	Induction of cellular senescence	(81, 86)
Omega-3 fatty acids (EPA and DHA)	PTEN-mediated CSF1R inhibition	Underexpression of miR-21	Anti-proliferative and pro-apoptotic effects in BC cell lines	(86)
Indole 3 carbinol	AHR-mediated CD4+ T helper activation	Overepression of miR-212 and miR-132	Enhancement of anti-cancer immune response	(94, 95)

Bcl2 protein, B cell lymphoma 2 protein; BC, breast cancer; MMPs, matrix metalloproteinases; EMT, epithelial-mesenchymal transition; PTEN, phosphatases and tensin homolog; FOXO3, forkhead box 3 protein; EF1A2, elongation factor 1A2; CSC, cancer stem-like cell; PCNA, proliferating-cell nuclear antigen; 1,25D, 1,25-dihydroxycholecalciferol vitamin D; ERα, estrogen receptor-α; SCFAs, short-chain fatty acids; FFARs, free fatty acid receptors; EPA, eicosapentaenoic acid; DHA, docosahexaenoic acid; CSF1R, colony stimulating factor 1 receptor; AHR, aryl-hydrocarbon receptor.

Polyphenols are a large family of natural compounds widely distributed in plant foods and have been shown to modulate, both *in vitro* and *in vivo*, the activity of several enzymes involved in the DNA metabolism (i.e. DNA methyltransferases, and histone deacetylases) (96). In this group, curcumin and curcuminoids have been assiduously studied as anti-inflammatory and anticancer agents (86, 87).

Curcumin (dyferuloylmethane) plays an onco-suppressor role by inhibiting several oncogenic pathways (88). *In vitro*, curcumin shows an anti-proliferative effect on cancer cell lines even through the modulation of expression of several miRNAs. For example, *in vitro*, curcumin induces the overexpression of the tumor suppressors miR-15a and miR-16, thus inhibiting some anti-apoptotic proteins (89). In MDA-MB-231 cells, curcumin upregulates miR-181b, interfering with the capacity of invasion and with the inflammation related to chemokines (90). In addition, it reduces the expression levels of genes involved in epithelial-mesenchymal transition (EMT) and invasion by controlling miR-34a expression in MCF-10F and MDA-MB-231 lines (91). Despite several studies suggest the anticancer activity of curcumin, its potential use is limited by peculiar pharmacodynamic properties: it has poor absorption, low serum levels, rapid hepatic metabolism, limited tissue distribution and short half-life (96).

Flavonoids (genistein, glabridin, glyceollins) and stilbenes (resveratrol) are polyphenols that affect the epigenetic regulation of genes involved in BC progression and drug-resistance. In TNBC cell lines, such as Hs578t, genistein suppresses miR-155 expression and up-regulates the expression of miR-23b, that has a pro-apoptotic and antiproliferative role (97).

Resveratrol treatment inhibits the proliferation on MCF-7 BC cells by upregulating miR-663, miR-141, miR-774, thus leading to the inhibition of elongation factor 1A2 (EF1A2) (85). In MDA-MB-231 cell lines, resveratrol exhibits strong anti-oxidant activity and induces apoptosis by increasing the levels of tumor-suppressive miRNAs, in particular miR-200c (87, 92).

The active metabolite of the vitamin D (1,25-Dihydroxyvitamin D3, Calcitriol) binds to the vitamin D receptor (VDR) and influences various signaling pathways involved in cell differentiation, cell cycle arrest and apoptosis (93). Furthermore, it regulates miR-182 expression, leading to protection of breast epithelial cells against cellular stress (81). Calcitriol also reduces the level of miR-489, which is an estrogen regulated miRNA promoting tumor cell growth induced by sexual hormones (98).

Other dietary components modulating miRNA expression in breast cancer cells are fatty acids (FAs) (81, 86) and indole alkaloids such as indole 3-carbinol (94, 95).

Several studies describe the role of physical activity and healthy diet in reducing breast cancer risk, also for subjects carrying *BRCA1/2* mutations (94, 99–101). In this context, a nutriepigenetic pilot study is being conducted to evaluate how a personalized nutritional and lifestyle intervention (NLI) can modulate expression of blood and salivary miRNAs associated with breast cancer risk in unaffected young women (<40 years) with *BRCA1/2* mutations (19, 102). This study also aims to evaluate whether NLI as primary prevention strategy may help deal with emotional distress occurring in women at high risk for hereditary breast and ovarian cancer who are involved in intensive screening programs.

## Conclusions

In this review, we have reported most of the published data evaluating the role of miRNAs in influencing BRCA-related breast cancer risk and diagnosis. Prospective validation of the reported results and standardization of miRNA isolation methods are, however, still awaited before their use in routine clinical practice. Interestingly, numerous preclinical and clinical studies have showed that *BRCA1/2* genes may interfere with and be silenced by several miRNAs. Furthermore, emerging evidences suggest the role of nutritional and lifestyle interventions in preventing breast cancer development, even through the modulation of breast cancer-related miRNAs. Prospective clinical trials evaluating the association between penetrance of BRCA mutations, NLI strategies for primary prevention and miRNA signatures are ongoing.

## Author Contributions

CT and BP contributed equally to the writing of the manuscript and tables. AM and DB were involved in planning and supervised the study. ASi, MMi, VU, BB, FB, PM, SP, ASq, MV, DC, GM, and MMe reviewed this work. All authors contributed to the article and approved the submitted version.

## Funding

This study was supported by a research grant from the Lega Italiana per la Lotta contro i Tumori (LILT) – bando di ricerca sanitaria 2017 (cinque per mille anno 2015) and by the Gruppo Oncologico Italiano di Ricerca Clinica (GOIRC).

## Author Disclaimer

Any views, opinions, findings, conclusions, or recommendations expressed in this material are those solely of the authors and do not necessarily reflect those of ESMO or Roche.

## Conflict of Interest

The authors declare that the research was conducted in the absence of any commercial or financial relationships that could be construed as a potential conflict of interest.

## Publisher’s Note

All claims expressed in this article are solely those of the authors and do not necessarily represent those of their affiliated organizations, or those of the publisher, the editors and the reviewers. Any product that may be evaluated in this article, or claim that may be made by its manufacturer, is not guaranteed or endorsed by the publisher.
